# A genome-wide association study identifies distinct variants associated with pulmonary function among European and African ancestries from the UK Biobank

**DOI:** 10.1038/s42003-023-04443-8

**Published:** 2023-01-14

**Authors:** Musalula Sinkala, Samar S. M. Elsheikh, Mamana Mbiyavanga, Joshua Cullinan, Nicola J. Mulder

**Affiliations:** 1 Computational Biology Division, Faculty of Health Sciences, Institute of Infectious Disease and Molecular Medicine, University of Cape Town, Anzio Rd, Observatory, 7925 Cape Town South Africa; 2 Pharmacogenetics Research Clinic, Campbell Family Mental Health Research Institute, Centre for Addiction and Mental Health, Toronto, ON Canada

**Keywords:** Data integration, Genome-wide association studies

## Abstract

Pulmonary function is an indicator of well-being, and pulmonary pathologies are the third major cause of death worldwide. We analysed the UK Biobank genome-wide association summary statistics of pulmonary function for Europeans and individuals of recent African descent to identify variants associated with the trait in the two ancestries. Here, we show 627 variants in Europeans and 3 in Africans associated with three pulmonary function parameters. In addition to the 110 variants in Europeans previously reported to be associated with phenotypes related to pulmonary function, we identify 279 novel loci, including an *ISX* intergenic variant rs369476290 on chromosome 22 in Africans. Remarkably, we find no shared variants among Africans and Europeans. Furthermore, enrichment analyses of variants separately for each ancestry background reveal significant enrichment for terms related to pulmonary phenotypes in Europeans but not Africans. Further analysis of studies of pulmonary phenotypes reveals that individuals of European background are disproportionally overrepresented in datasets compared to Africans, with the gap widening over the past five years. Our findings extend our understanding of the different variants that modify the pulmonary function in Africans and Europeans, a promising finding for future GWASs and medical studies.

## Abstract (web summary)

A genome-wide association study using summary statistics from the UK Biobank identifies ancestry-specific variants associated with pulmonary function among European and African ancestry cohorts.

## Introduction

Pulmonary function measures using the spirometer are indicators of respiratory health and predict morbidity and mortality^1,2^. However, these parameters, which include the force expiratory volume in 1-second (FEV1), forced vital capacity (FVC), and peak expiratory capacity (PEF), vary significantly among populations of different ancestry backgrounds^3^ and show strong evidence of genetic and environmental influences^1,4^.

During the last decade, large-scale genome-wide association studies (GWASs) have used various pulmonary parameters to evaluate the genomic loci associated with pulmonary function and related traits that have yielded hundreds of associated variants^5–10^. These and other studies indicate that genomic loci associated with pulmonary function overlap with chronic obstructive pulmonary disease, asthma, pulmonary fibrosis, lung cancer, and other pulmonary phenotypes^2,8–10^. For example, a recent GWAS based on the UK Biobank cohort (*N* = 50,008), including heavy smokers and never smokers, identified six loci associated with low FEV1^10^. Another study of individuals (*N* = 48,943) sampled from the extremes of pulmonary function distribution in the UK Biobank identified 95 variants strongly associated with chronic obstructive pulmonary disease susceptibility^8^. Importantly, these previous studies have applied the analyses to a selected population group of primarily European ancestry.

The UK Biobank cohort contains data on 389,449 individuals, providing an opportunity to use GWAS approaches to identify variants associated with pulmonary function among individuals of European and recent African descent by allowing large-scale comparisons of lung function parameters^11^. Furthermore, by integrating the genetic association of FEV1, PEF, and FVC, a list of shared loci that collectively modify pulmonary function could be identified. We hypothesise that different genetic variants are associated with pulmonary function in Africans. Thus, their identification will provide additional information relevant to understanding pulmonary function in physiology and disease in district populations. However, to our knowledge, no GWAS study has been performed to compare the SNPs associated with the full range of FEV1, FVC, and PEF parameters across the entire UK Biobank cohort and separately among Africans and Europeans.

Here, we compare variations in pulmonary function parameters among individuals of African and European ancestry represented in the UK biobank. First, we used the genome-wide associated summary statistics for three UK Biobank-defined continuous pulmonary function parameters: FEV1, FVC, and PEF. Then, we conducted further analyses to identify genes, regions, and gene sets associated with each pulmonary phenotype. Furthermore, we evaluate the candidate phenotype variants in relation to published GWAS results. Overall, this approach allows us to report credible loci associated with pulmonary function among Africans and Europeans, which were enriched across many plausible genes and gene sets involved in pulmonary function or related phenotypes.

## Results

### UK Biobank pulmonary function demographics

There were 389,449 participants, comprising Europeans (*N* = 383,471) and Africans (*N* = 5978). The average participant age at recruitment was 56.8 years (standard deviation = 8.0 years) for Europeans and 51 years (7.9) for Africans, respectively. This difference was statistically significant (Welch test: *t* = −41.3, *p* = 9.07 × 10^−300^) (see Supplementary Fig. 1).

### Lung function parameters vary between individuals of European and African ancestry

**Fig. 1:**
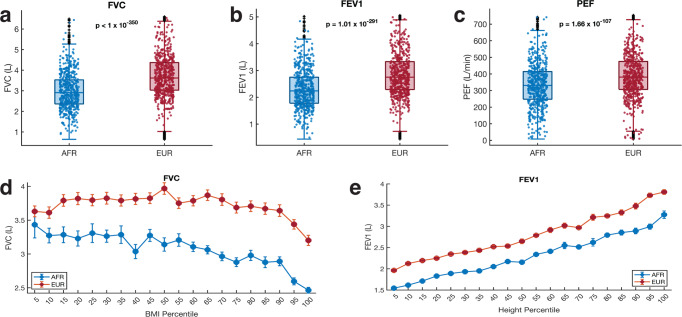
Comparison of the pulmonary function parameter among Africans and Europeans. The boxplots indicate the distribution of (**a**) FEV1, (**b**) FVC, and (**c**) PEF in Europeans (*n* = 383,471) and Africans (*n* = 5978). The *p*-values shown for each comparison were calculated from Welch’s *t*-test. On each box, the central mark indicates the median, and the left and right edges of the box indicate the 25th and 75th percentiles, respectively. The whiskers extend to the most extreme data points not considered outliers, and the outliers are plotted individually using the ‘+‘ symbol. To make the visualisation clearer, the filled circle mark showing the distribution only includes 1000 randomly sampled points from the total sample size of each group. Error bars showing the variation in (**d**) FVC and (**e**) FEV1 across BMI percentiles and height, respectively, among Africans and Europeans. The middle point indicates the mean FVC or FEV1/FVC, and the error bars indicate the standard error of the mean at the BMI percentile.

We assessed the mean FVC, FEV1, and PEF between Europeans (*N* = 383,471) and Africans (*N* = 5978) represented in the UK Biobank datasets. We found that the mean FVC was significantly higher in the Europeans (mean = 3.73 L) compared to the Africans (mean = 2.95 L), (Welch test: *t* = 48.35, *p* < 1 × 10^−320^; Fig. 1a). Furthermore, we found that the FEV1 and the PEF were both significantly higher in Europeans (mean FEV1 = 2.82 L, mean PEF = 389.6 L/min) than those measured in the Africans (mean FEV1 = 2.28 L, mean PEF = 332.7 L/min), FEV1; *t* = 42.60, *p* = 1.0 × 10^−291^ (Fig. 1b) and PEV; *t* = 24.06, *p* = 1.7 × 10^−107^; Fig. 1c. According to a recent systematic review, “Whites” have higher pulmonary function parameters than other ethnic groups (including Africans)^12^. About 50% of these articles cited inherent factors and anthropometric differences to explain the observed differences. However, similar to other studies^13–15^, our findings show that these variations in pulmonary function measures exist across various ages, heights, and BMI percentiles (Fig. 1 and Supplementary Fig. 2a–i). However, using a generalised linear model, we found that the observed higher FVC, FEV1, and PEF in Europeans compared to Africans is not due to the age difference between the two groups, even though the FVC (*t* = −19.26, *p* = 1.0 × 10^−82^), FEV1 (*t* = −16.68, *p* = 1.88 × 10^−62^), and PEF (*t* = −11.91, *p* = 1.01 × 10^−32^), tend to reduce with age (see Supplementary Table 1 and Supplementary Note 1). Recently, a lack of knowledge among healthcare workers concerning variations in pulmonary function measures among ethnic groups has been suggested to impact the assessment of minority patients’ recovery from COVID-19^15^. However, no studies have identified major genetic variants that vary by ethnic groups that can explain the disparities in lung function^15,16^.

Previous studies show that the FVC, FEV1, and PEF vary with age, body mass index (BMI), and height of individuals^17–20^. Here, we also found that FVC, FEV1, and PEF tend to reduce with age, an increase in BMI is observed at the 50 percentile, and all three parameters increase along with the height of the individuals (Fig. 1d,  e, and Supplementary Fig. 2a, i). However, unlike age and height, we found that the relationship between pulmonary function parameters and BMI appears to be associated with overweight/obesity, with a threshold effect and not a simple linear relationship (see Supplementary Note 1). Furthermore, we observed that the FEV1/FVC levels are conversely higher in Africans than Europeans across the BMI percentiles (Supplementary Fig. 2f).

### Genetic variant associated with FVC, FEV1 and PEF among Europeans and Africans

Since the FVC, FEV1, and PEF values were significantly higher in Europeans than in Africans, we presumed that a genome-wide association analysis would identify the genetic variants associated with each of these pulmonary function parameters in each group. Therefore, we collected the GWAS summary statistics for each pulmonary function parameter within each ethnic group (see the “Methods” section). In these data, we discovered 1 variant in Africans and 67,855 variants in Europeans that were associated (GWA *p*-values 5 × 10^−8^) with FEV1, 6 in Africans and 79,132 in Europeans that were associated with FCV, and zero (0) in Africans and 26,432 in Europeans that were associated with PEF (Supplementary Fig. 3a–c). The total number of significant variants discovered for each pulmonary function parameter, including those in substantial linkage disequilibrium (R^2^ > 0.4), and the intersection of these variants are displayed in Supplementary Fig. 3a–f.

**Fig. 2:**
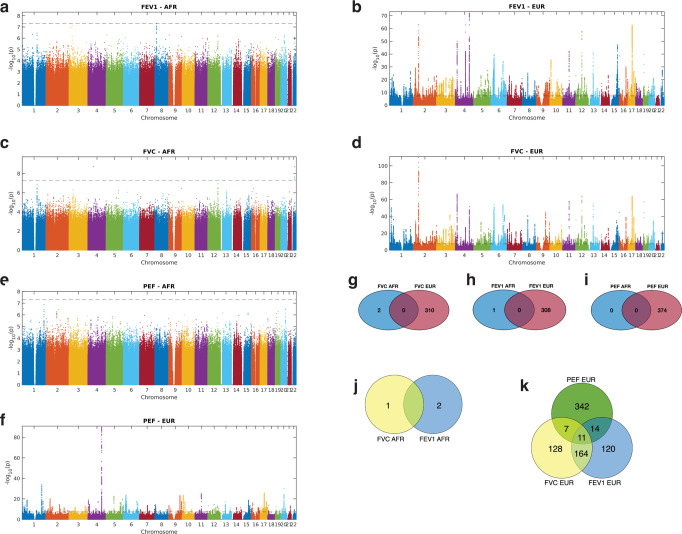
Manhattan plots and Venn diagrams of the SNP associations. The Manhattan plots include those of the SNPs associated with (**a**) FEV1 in Africans and (**b**) FEV1 in Europeans, (**c**) FVC in Africans and (**d**) FVC in Europeans, and (**e**) PEF in Africans and (**f**) PEF in Europeans for each chromosome. The Venn diagrams show the overlap among the significant causal SNPs associated with (**g**), FVC (**h**), PEF, and (**i**) FEV1 in Africans and Europeans. The distribution of genetic variants associated with three pulmonary function parameters among the (**j**) Africans and (**k**) Europeans. Refer to Supplementary Data 1 for details concerning individual SNPs and their frequencies among Africans and Europeans.

We applied fine mapping^21^ to identify 310 (credible set) casual variants significantly associated (*p*-values < 5 × 10^−8^ and causal probability >0.1; see “Methods” section) with FVC in Europeans and 2 significant associations in Africans (Fig. 2a, b). For FEV1, we found 308 significant causal variant associations in Europeans and 1 in Africans (Fig. 2c, d). Furthermore, for PEF, we identified 374 significant causal variant associations in Europeans and none (0) in Africans (Fig. 2e, f). Overall, we identified 820 unique credible SNPs associated with the three pulmonary functions. Surprisingly, the significant SNPs associated with FVC, FEV1, and PEF were unique to each ancestral group (Fig. 2g–i and Supplementary Data 1).

Next, we evaluated the independent SNPs associated with the three pulmonary function parameters while considering the population’s linkage disequilibrium structure (see the “Methods” Section). Here, we identified 630 independent SNPs from the 820 credible sets of causal SNPs associated with all three pulmonary function parameters. Finally, we compared the 627 independent SNPs in Europeans with the 3 SNPs in Africans significantly associated with the three pulmonary function parameters and found no common variants between the two sets. Conversely, we found that 164 SNPs were associated with FVC and FEV1 in Europeans (Fig. 2k). However, there was no overlap in the associated SNPs among Africans (Fig. 2j). Finally, it should be noted that smoking impacts pulmonary function, but the effect of smoking was not accounted for in the GWA analyses. Therefore, this is probably a limitation of our findings.

Since the SNPs significantly associated with pulmonary function were unique for Europeans and Africans, we next relaxed the GWAS significance threshold to a suggestive cut-off *p*-value^22^ of 1 × 10^−6^. Then, we compared the significant SNPs in Europeans and Africans for FVC, FEV1, and PEF. For all three pulmonary function metrics, even when using a less strict significance criterion, we were unable to discover any shared SNPs between Africans and Europeans (Supplementary Fig. 3d–f). Furthermore, we found that the most statistically significant SNPs in Africans had relatively larger beta estimates in Africans than Europeans for the FCV, FEV1, and PEF (see Supplementary Fig. 4 and Supplementary Notes 2). In addition, we have provided an interactive online visualisation that allows the user to evaluate the significance of SNPs in each group using an arbitrary significance threshold and compare the SNPs on different chromosomes, linkage disequilibrium loci, and genes, for FVC (Supplementary Figs. 5,  6), FEV1, and PEF (see the Supplementary Notes: Comparison of variants associated with pulmonary function).

We compared the minor allele frequency of SNPs in the UK Biobank between Europeans and Africans for the combined 820 SNPs (817 in Europeans plus 3 in Africans) associated with pulmonary function. We found that 788 out of 820 SNPs differed significantly in frequency between Africans and Europeans (Supplementary Data 2). The top-three variants that exhibited the most significantly higher frequencies in Europeans compared to Africans were rs2042395 (frequency in Europeans = 0.77, In Africans = 0.19, Fisher test *p*-value = 4.94 × 10^−323^), rs3748400 (Europeans = 0.78, Africans = 0.19, *p* = 6.92 × 10^−323^), rs8045843 (Europeans = 0.78, Africans = 0.17, *p* = 8.89 × 10^−323^), see Supplementary Data 2 and Supplementary Fig. 2g. Interestingly, the variants rs2042395 and rs8045843 have been previously associated with the “well-being spectrum”^23^ and “sensitivity to environmental stress and adversity”^24^, respectively, in individuals of European ancestry. Conversely, the top variants with higher frequency in Africans compared to Europeans were rs143384 (Europeans = 0.40 and Africans = 0.92, *p* = 2.0 × 10^−323^), rs3133084 (Europeans = 0.23 and Africans = 0.65, *p* = 8.4 × 10^−323^), and rs7853063 (Europeans = 0.20 and Africans = 0.60, *p* = 6.4 × 10^−323^), see Supplementary Fig. 2g. Among these, the variant rs143384 has been reported to be associated with FVC, lung function, and PEF^25^, and among anthropometric traits in Europeans^26^.

Altogether, these analyses revealed that different SNPs may be associated with FVC, FEV1, and PEF among Europeans and Africans and that the frequency of these SNPs significantly varies between these populations.

### Pathway and GWAS catalog enrichments of the SNPs

We assessed the enrichment of GWAS Catalog^27^ annotation terms for the genes containing SNPs associated with lung function (suggestive cut-off *p*-value of 1 × 10^−6^) in each study population (see Supplementary Data 2).

**Fig. 3:**
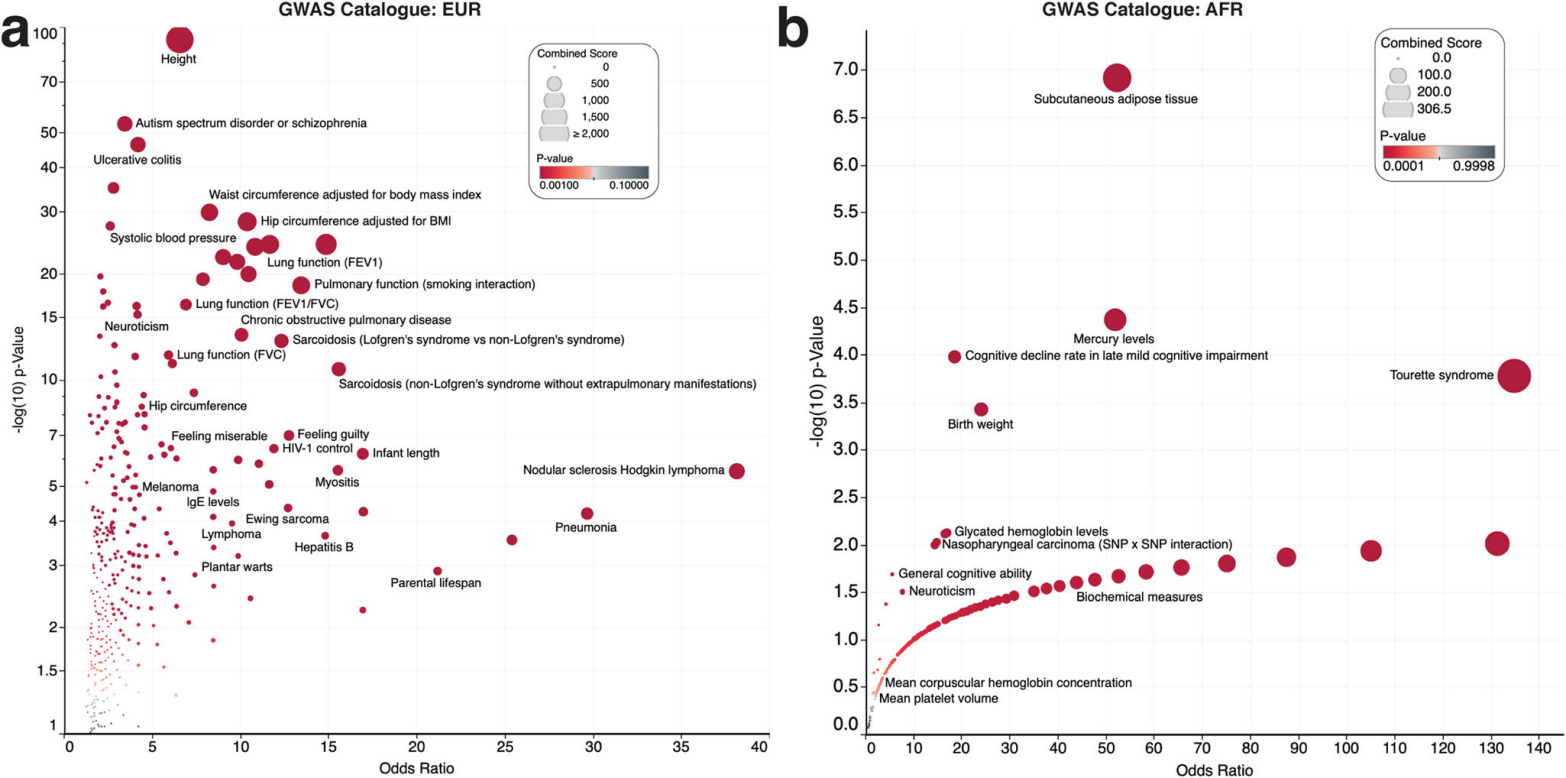
GWAS catalog enrichment analysis plots. Volcano plots of the GWAS Catalog enrichment analysis for genes where significant SNPs are located for (**a**) Europeans and (**b**) Africans. All four plots show the adjusted *p*-value on the *y*-axis and the odds ratio of the enrichment score on the *x*-axis. Each circle represents a GWAS Catalog term or Elsevier pathway. The circles are coloured based on the levels of statistical significance, with the redder colours showing a greater degree of significance. Each circle is sized based on the combined enrichment score of the term represented by the circle.

The GWAS Catalog term analyses revealed that in Europeans, the genes were significantly enriched for GWAS terms associated with “Height” (hypergeometric test; *p* = 1.06 × 10^−93^), “Lung function (FEV1)” (*p* = 5.4 × 10^−25^), “Pulmonary function interaction” (*p* = 2.33 × 10^−19^) among others (Fig. 3a and Supplementary Data 3). In Africans, we found that the genes were significantly enriched for GWAS terms associated with “Subcutaneous adipose tissue” (*p* = 1.2 × 10^−07^), “Birth weight” (*p* = 3.7 × 10^−04^), “Cognitive decline rate in late mild cognitive impairment” (*p* = 7.3 × 10^−04^), among others (Fig. 3b and Supplementary Data 3). Overall, these results show that the SNPs identified among Europeans are in genes known to play roles in many phenotypes, most notably those related to pulmonary function or GWAS phenotypes related to pulmonary function. Conversely, the SNPs we identified associated with pulmonary function among Africans fall within genes that are not enriched for pulmonary function-related terms.

### Variant spanning loci associated with pulmonary function among Europeans and Africans

**Fig. 4:**
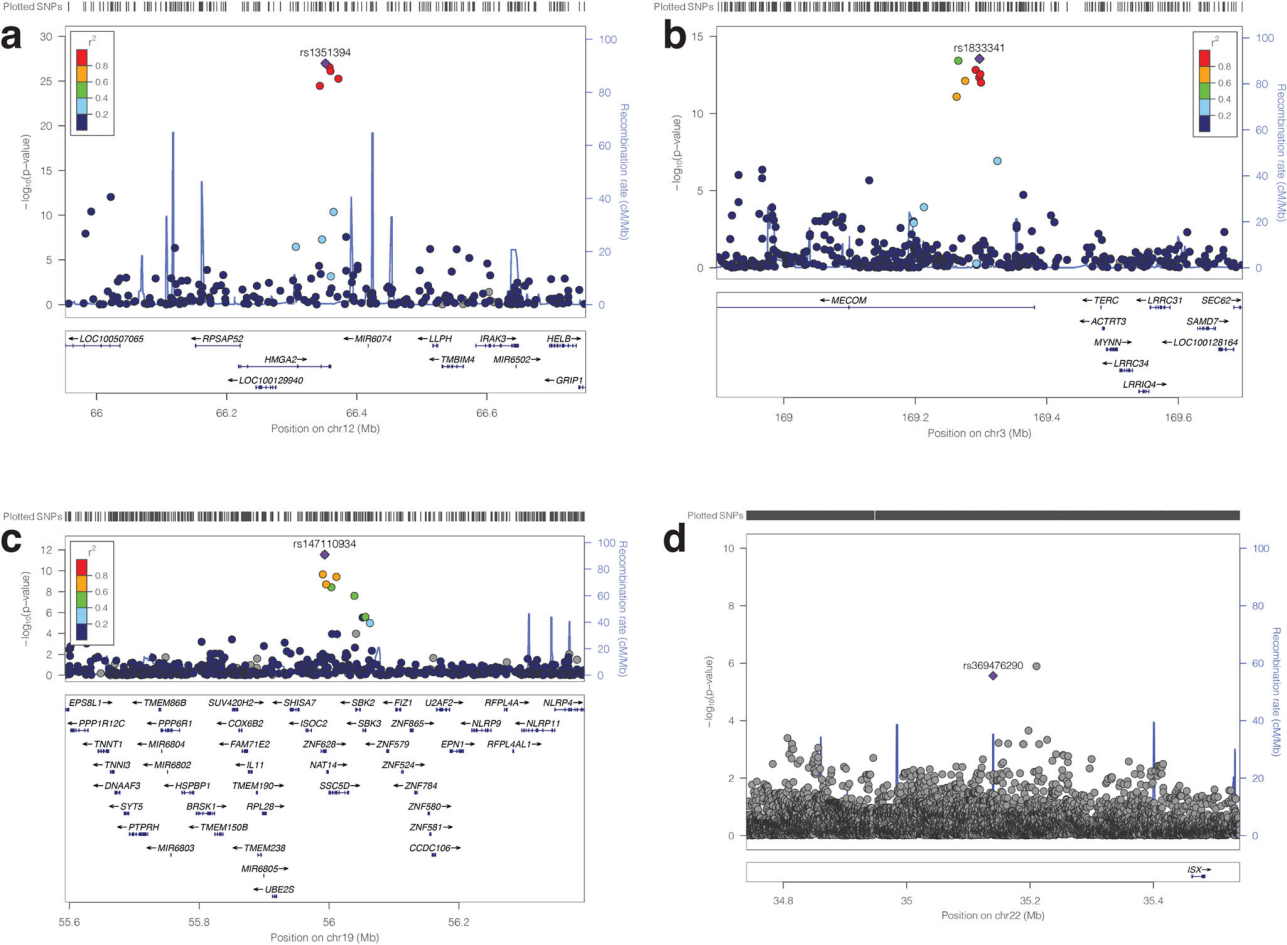
Regional association plots for genome-wide significant pulmonary function. These include the loci for the lead SNPs (**a**) rs1351394 at loci 12q14.3, (**b**) rs16909898 at 9q22.32, (**c**) rs147110934 at loci 19q13.42, and (**d**) rs369476290 on chromosome 22. The genes within the chromosomal loci are shown in the lower panel. The blue line indicates the recombination rate. The filled circles show the position of the SNPs along the region on the *x*-axis and the negative logarithm of the association *p*-value on the *y*-axis. The lead SNP is shown in purple, and the SNPs within the locus are coloured based on the linkage disequilibrium correlation value (r2) with the lead SNP based on the European HapMap haplotype (in panels **a**, **b**, and **c**) and African HapMap haplotype (panel **d**) from the 1000 genome project.

Many of the associated SNPs may simply reflect the linkage disequilibrium structure of the populations^28,29^ (see Supplementary Data 4). For example, we found 10 variants associated with FEV1 and FVC in Europeans within loci 12q14.3, and upon fine mapping^21^, we found that the most likely causal SNP within the loci was rs1351394 (Probabilistic Identification of Causal SNPs^21^, causal probability value = 0.7243), a 3-prime untranslated region variant located in the gene *HMGA2* (Fig. 4a). The variant rs1351394 has previously been associated with variations that affect FEV1 capacity, including height^30,31^ and birth length^32^. Furthermore, *HMGA2* is involved in lung development^33^.

At locus 19q13.42, we found that the most likely causal SNP is rs147110934 (causal probability = 0.83), associated with FEV1 and FVC in Europeans (Fig. 4b, also see Supplementary Data 4). rs147110934 is a predicted missense variant that falls within the *ZNF628* gene. In addition, whilst rs147110934 has not been previously associated with pulmonary function, we found it is associated with height^34^ and body weight^35,36^, both of which are associated with FVC and FEV1.

Furthermore, we found several SNPs in the loci 9q22.32 associated with pulmonary function (Fig. 4c). Here, the lead and predicted causal (causal probability = 1) variant is rs16909898, located in the *PTCH1* gene previously identified to modify pulmonary function parameters^37,38^ and height^31^.

In addition, for individuals of African ancestry, at the locus 5q32, the lead SNP among the four associated with pulmonary function was rs369476290 (causal probability = 0.67), an intergenic variant located near the gene *ISX*. rs369476290 has not been previously linked to pulmonary function or disease (Fig. 4d).

Since the SNPs significantly associated with pulmonary function were unique for Europeans and Africans, we next set to compare the estimated beta values for all SNPs with a GWA significance of <0.05. Here, we found that the most statistically significant SNPs in Africans had relatively larger beta estimates in Africans than Europeans for the FCV, FEV1, and PEF (Supplementary Fig. 4). Overall, this finding showed that the SNPs significantly associated with pulmonary function in Africans demonstrated larger effect sizes than in Europeans. Conversely, we found thousands of variants associated with pulmonary function in Europeans that tended toward statistical significance in Africans (see Supplementary Notes: Comparison of variants associated with pulmonary function).

Furthermore, we aimed to replicate the causal variants associated (*p* < 5 × 10^−8^) with pulmonary function in Europeans in Africans at a *p*-value of less than 0.05. Interestingly, we found 56 independent variants that could be associated with pulmonary function in both Europeans and Africans (see Supplementary Note 3). These include, among others, the loci near the gene *MECOM*, where the causal SNP rs11709963 was associated with FEV1 (*p*-value = 5.3 × 10^−19^) in Europeans. There was some evidence for an association within the region for Africans (rs1362771, r^2^ = 0.51 the causal SNP rs11709963 in Europeans) was associated with FVC (replication *p* = 0.02), see Supplementary Data 4 and Supplementary Fig. 7. Furthermore, a *SATB2* variant, rs77064030 (*p*-value in Europeans = 6.7 × 10^−11^) that is in linkage disequilibrium with rs78696503 (r^2^ = 0.8), associated with FEV in Africans (replication *p*-value in Africans = 0.007), see Supplementary Fig. 8. Among variants associated with PEF, is the *FAM132A* variants rs79361800 (*p*-values; Europeans = 9.20 × 10^−10^ and Africans = 1.02 × 10^−5^), see Supplementary Fig. 9.

Therefore, we suggest that our findings may be due to both the difference in the sample size (which is associated with the statistical power to identify the causal variants) and the existence of different variants associated with pulmonary function among European and African individuals.

### Comparison to variants previously associated with pulmonary function

Next, we aimed to identify the previously described and novel SNPs among the significant SNPs that were also predicted to be causal within a particular linkage disequilibrium block (see the “Methods” section). Here, we grouped the SNPs into four ordinal categories based on confidence: (1) SNPs reported to be associated with pulmonary function, (2) SNPs related to phenotypes correlated to pulmonary function (e.g., height, see Supplementary Fig. 1), (3) SNPs that fall within genes reported to be associated with pulmonary function and/or disease, (4) SNPs that are expression quantitative trait loci (eQTLs) in the lung, and (5) the novel SNPs.

Interestingly, we found that among our list, 97 variants in Europeans and none (0) in Africans have been previously associated with pulmonary function (see Table 1 and Supplementary Data 4). These include variants in the genes *PLEKHM1*, *HMGA2, KDM2A*, and *SYTL2* (Table 2). Likewise, we found that 69 variants in Europeans, and none (0) of the variants in African ancestry individuals had previously been associated with a phenotype correlated to pulmonary function (see Supplementary Data 4). Furthermore, we found that 178 variants in Europeans and 0 variants in Africans are located within genes associated with various pulmonary function phenotypes and diseases, and 4 variants in Europeans and none in Africans are significant eQTLs in the lungs. These four variants affect the expression of *CAMLG*, *PHF15*, *RNF40*, and *MLLT6*. Finally, we found 206 novel variants in Europeans and 3 in Africans associated with pulmonary function; see Supplementary Data 4 for the complete list of significant variants and the studies reporting the known variants. Among the novel discoveries, in Europeans, 104, 101 and 136 were associated with FVC, FEV1, and PEF, respectively, whereas in Africans, 2, 1 and 0 were associated with FVC, FEV1, and PEF, respectively.

**Table 1:** Known and novel variants associated with pulmonary function.

Ethnicity	Pulmonary Function	Pulmonary Function Associated Traits	Lung Disease Associated	eQTLs*	Novel
Africans	0	0	0	0	3
Europeans	97	69	178	4	279

^*^Expression quantitative trait loci.

**Table 2:** Top significant variants associated with pulmonary function.

Variant	Nearest Genes	Ethnicity	Measure	Chrom:Pos	GWAS p	Evidence
rs536516159	*LZTS1*	AFR	FEV1	8: 20598779	4.8 × 10^−08^	Novel
rs8756	*HMGA2, AC090673.2*	EUR	FEV1	12: 66359752	8.4 × 10^−58^	Pulmonary Function
rs55663797	*PLEKHM1*	EUR	FEV1	17: 43544379	7.3 × 10^−52^	Lung Disease Assoc.
rs1828591	*HHIP*	EUR	FEV1	4:145480780	8.3 × 10^−52^	Pulmonary Function
rs571481915	*LPHN3*	AFR	FVC	4: 61183218	1.8 × 10^−09^	Novel
rs369476290	*ISX*	AFR	FVC	22: 35140134	2.0 × 10^−09^	Novel
rs8756	*HMGA2*	EUR	FVC	12: 66359752	1.2 × 10^−63^	Pulmonary Function
rs2696624	*KANSL1*	EUR	FVC	17: 44326845	1.8 × 10^−62^	Novel
rs7952436	*KDM2A, ADRBK1*	EUR	FVC	11: 67024534	9.3 × 10^−58^	Pulmonary Assoc.
rs6829956	*HHIP*	EUR	PEF	4:145440288	1.1 × 10^−90^	Pulmonary Function
rs1342062	*SLC26A9*	EUR	PEF	1:205912786	1.6 × 10^−34^	Lung Disease Assoc.
rs143384	*GDF5*	EUR	PEF	20: 34025756	5.8 × 10^−31^	Pulmonary Function

We focused on the genes in which the novel SNPs associated with pulmonary function among Europeans were located to perform enrichment analyses based on the Disease Gene Network database^39^, and the Phenotype and Genotype Integrator database^40^. Here, our Disease Gene Network analysis revealed that the novel genes are enriched for terms related to pulmonary function, including “Forced expiratory volume function” (*p* = 9.7 × 10^−13^) and body measures that modify pulmonary function, including “Body Height” (*p* = 1.33 × 10^−15^), see Supplementary Fig. 10. Similarly, our phenotype and genotype integrator enrichment analysis revealed that the genes are enriched for pulmonary function-related terms, including Forced Expiratory Volume (*p* = 2 × 10^−4^) and phenotypes associated with pulmonary function, including Body Height (*p* = 4.2 × 10^−07^), see Supplementary Fig. 10. These findings show that despite the SNPs being novel among Europeans, the genes within which the SNPs are located are known to be associated with pulmonary function.

### Bias in GWAS studies explains why few SNPs were previously associated with pulmonary function in Africans

**Fig. 5:**
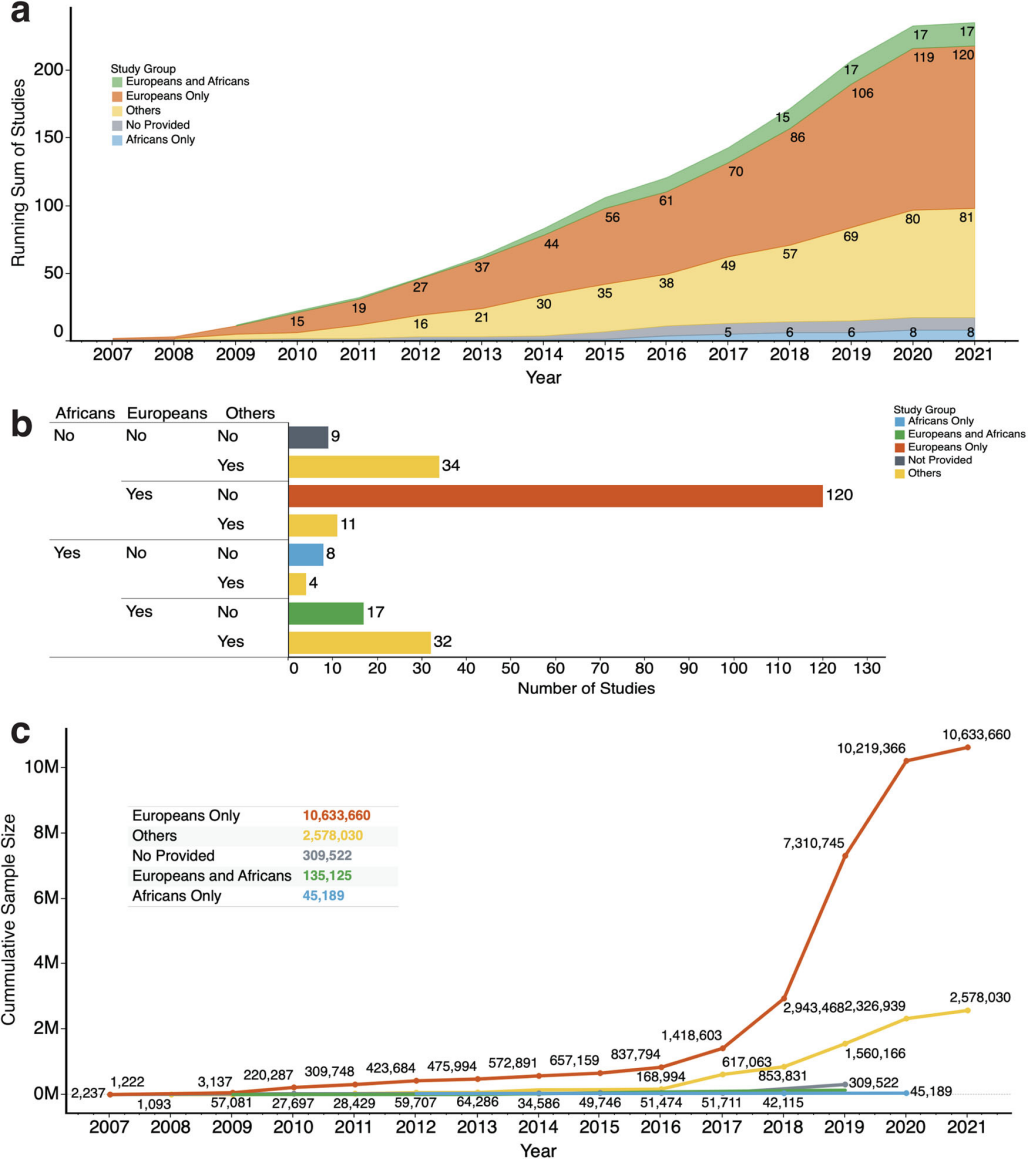
The GWAS catalog of pulmonary function and lung phenotypes studies. **a** The plot of the running sum of GWAS studies reported from 2007 to 2021. The colours show details about the race/ancestry groups: Africans only, Europeans only, Europeans and Africans, Others, and those for which the race/ancestry group is “Not provided”. **b** The total number of GWAS studies reported for each race/ancestry group combination. The colours depict information about race and ethnic groups. **c** The trend of the cumulative sum of participants (on the *y*-axis) of studies from 2007 to 2021. The colours show details about the race/ancestry groups. The marks are labelled by the cumulative sum of participants. The figure insert shows the total number of participants by race/ancestry group.

Since none of the SNPs we identified as being associated with pulmonary function among Africans has been reported in the literature, we queried the GWAS Catalog^27^ for previous studies of pulmonary function or phenotypes related to pulmonary function (such as asthma) across various ancestry backgrounds. We found those studies to be significantly biased toward individuals of European ancestry (Fig. 5a). Also, despite the number of studies conducted on individuals of African ancestry increasing over the last five years, the gap is widening between the number of studies reported on Europeans compared to Africans during the same time interval (Fig. 5a). Overall, among the 235 GWAS studies reported on pulmonary function or phenotypes related to pulmonary function, only eight were conducted on Africans or African Americans. In comparison, we found that 120 studies have been conducted exclusively on individuals of European ancestry (Fig. 5b). Furthermore, in the same studies, the cumulative sample size of the Europeans in 2021 (10,633,660 individuals) is approximately 235 times greater than that of the Africans (45,189 individuals; see Fig. 5c).

## Discussion

We analysed variations in pulmonary function and the associated genetic variants among individuals of African and European ancestry in the UK Biobank. Here, we report differences in FEV1, FVC, and PEF parameters among Africans and Europeans. Previous studies have examined the pulmonary function parameters between Africans and Europeans, with most reporting the differences we observed^3,41–43^. However, there has been no explanation for the genetic basis of these observed differences.

Here, we showed that the SNPs associated with pulmonary function differed between Europeans and Africans. Others have reported that the genetic variants associated with various phenotypes may differ among individuals of different ancestry^44–47^. For example, we found that the SNPs associated (*p* < 5 × 10^−8^) with pulmonary function in African individuals were non-significant in Europeans, even at a p-value cut-off threshold of 0.05 (see Supplementary Note 2). Our findings confirmed that different variants might be associated with pulmonary function among Africans and Europeans. Despite this observed difference between the two ancestral groups, we are also cognizant that the number of individuals of African ancestry represented in the UK Biobank is much lower than that of Europeans. To some extent, the smaller calculated beta estimates with larger standard errors in the African group compared to the European group are explained by the difference in the sample size (see the interactive plot here). Therefore, the smaller sample size of Africans may have resulted in us missing some common associations among the groups^48,49^. It would be interesting to evaluate our findings based on a larger sample of individuals of African ancestry.

Given that the frequency of SNPs, primarily those we found associated with pulmonary function, varies between Africans and Europeans, it is apparent why different variants are associated with these traits^48^. For example, we found that rs12925700 is approximately 21 times more frequent, and rs11205303 is 14 times more frequent in Europeans than Africans, and both SNPs are reported elsewhere^50,51^ and here as being associated with pulmonary function in Europeans. Furthermore, the frequency of genetic variants among individuals of a particular ancestry affects the penetrance of disease and phenotype associated with the alternate alleles^48,52–55^. For example, non-alcoholic fatty liver disease^56^, serum uric acid levels^57^, white blood cell count^58^, fatty acid desaturases^59^, and other phenotypes^60–62^ are associated with different alleles among Africans and Europeans. These alleles are sometimes located on the same gene, but their frequencies vary between ancestral groups.

Our enrichment analyses demonstrated a link between the significant SNPs and GWAS Catalog terms associated with pulmonary function in Europeans, with several results showing plausible biological mechanisms. Whereas it was apparent that the significantly enriched terms in Europeans were mainly associated with pulmonary function and related phenotypes (Fig. 3), we found that the top-ranking terms among SNPs in Africans are not related to pulmonary function. This finding exemplifies the bias in previous GWAS studies that have not picked up genes associated with pulmonary function in Africans. We believe that more GWAS on larger groups of Africans than those presented here are needed to identify the variants that modify pulmonary function and other traits.

We also showed that genetic association studies of pulmonary function, pulmonary physiology, and pathology are significantly biased toward individuals of European ancestry. Even in cases where individuals of African ancestry are included in the studies or studied separately, the number of participants is lower than that of individuals of European ancestry. Furthermore, the trend shows that this gap has widened vis-à-vis how Africans and Europeans are studied over the last few years (see Fig. 5).

In summary, we have revealed the extent of variations between Africans and Europeans in the pulmonary function parameters: FEV1, FVC, and PEF. In addition, we have identified the different genetic variants associated with pulmonary function among individuals of African and European ancestry. Our integrative analysis of the causal genetic variants, together with the GWAS phenotypes and diseases associated with the genes in which the variants fall, indicates that the significant SNPs are associated with pulmonary function and related phenotypes in Europeans. Therefore, more genetic association studies focusing on people of African ancestry are evidently needed to identify and validate additional causal variants for these traits and other diseases.

## Methods

We analysed a UK Biobank^11^ dataset of 383,471 individuals of European ancestry (designated as White, British, Irish, and “any other white background”) and 5978 individuals of recent African ancestry. The UK biobank obtained all participant samples and body measurements from consenting individuals. Information on the UK biobank ethics policy and approval can be found here: https://www.ukbiobank.ac.uk/learn-more-about-uk-biobank/about-us/ethics.The demographics of the UK Biobank participants are extensively described elsewhere^11^. The data elements we analysed include genotyping array data of imputed SNPs, anthropometric measurements, and pulmonary function parameters: FVC, FEV1, and PEF. The ancestry groups were initially defined by self-identification. Then, a principal component analysis was performed, followed by a random forest on the projected principal component analysis data to reassign the initial self-defined ancestries of individuals with a membership posterior probability >0.5. Other individuals with a posterior probability less than 0.5 for any given ancestry group were dropped from further analysis.

### Comparison of pulmonary function parameters in Europeans and Africans

We compare the mean values of the pulmonary function parameters FVC, FEV1, and PEF between 383,471 Europeans and 5978 Africans using the Welch t-test. Furthermore, to evaluate how FVC, FEV1, PEF, and FEV1/FVC values vary with the participant’s body mass index, height, and age, we calculated the 10th percentile bins of each anthropometric measurement and visualised the trend using error bars plotted for each percentile.

### Genome-wide identification of genetic variants and associations

The methods applied for genotyping participants in the UK Biobank are reported elsewhere^11,63^. Furthermore, the genotyping quality control implemented for the analyses is described at the following link https://pan.ukbb.broadinstitute.org/docs/qc. We obtained the GWAS summary statistics computed by the UK Biobank project for each pulmonary function parameter. The methods used to perform the GWA analyses are described elsewhere^64,65^. Briefly, the GWAS was performed for the pulmonary function phenotypes and ancestry groups using the Scalable and Accurate Implementation of Generalized Mixed Model Approach 65, using a linear or mixed logistic model including a kinship matrix as a random effect and covariates as fixed effects. The covariates included the participant’s age, sex, age multiplied by sex, the square of the age, the square of the age multiplied by the sex, and the first 10 principal components calculated from the genotype datasets. The Manhattan plots were produced in MATLAB using the software described here^66^. Furthermore, we used the Probabilistic Identification of Causal SNPs software with default settings to fine-map SNPs to identify the most credible causal SNPs within each linkage disequilibrium block while conditioning on the lead SNP signal in each locus ±500 kb^21^.

### Identification of unique and common variants

We applied the following approach to identify the unique variants associated with pulmonary function traits in Africans and Europeans. First, we extracted all the credible sets of causal variants associated with pulmonary function (FVC, FEV, and PEF) within ±500 kb of the most statistically significant variant within a particular linkage disequilibrium block. Then, the linkage disequilibrium structure of the populations was estimated using the UK Biobank and the same individuals used in the analysis. If a causal variant associated with one pulmonary function parameter (e.g., FVC) was associated with another pulmonary function measure (e.g., *p*-values <5 × 10^−8^ for FEV1) or in linkage disequilibrium (r^2^ > 0.4) with a variant associated with another pulmonary function parameter (e.g., FEV1), then we return the most statistically significant variant (i.e., the variant with the smallest GWA estimated *p*-value). This approach allowed us to remove 190 non-independent variants from the 820 (FVC = 310, FEV1 = 309, and PEV = 374) causal variants, leaving 630 independent (credible set) causal variants (FVC = 256, FEV1 = 233, and PEF = 297) associated with pulmonary function.

### Replication of variants in Africans

We attempted to replicate the significant finding from Europeans in Africans because the variants associated (*p* < 5 × 10^−8^) with pulmonary function in Europeans were not associated with pulmonary function in Africans. Here, for variants significantly associated with a pulmonary function parameter, we extracted all the variants linked (linkage disequilibrium: r^2^ > 0.4) to the causal variant. The linked variants in Europeans were then assessed for their association with the trait in Africans by extracting the estimate of GWA p-values in Africans and adjusting the *p*-values using the Benjamini and Hochberg procedure. Finally, we considered all variants with the adjusted *p*-values <0.05, within each linkage disequilibrium block, as evidence of local replication.

### Pathways and enrichment analyses

We used NBCI’s dbSNP^67,68^ to ascribe the significant variants associated (suggestive cutoff *p*-value of 1 × 10^−6^)^22^ with pulmonary function identified using GWAS to specific genes. This yielded a list of genes associated with pulmonary function in Europeans or Africans. Finally, using these two gene lists (for Europeans and Africans), we separately performed gene set enrichment analysis^69^ using Enrichr^70^ to identify the Elsevier pathways^70^, Disease Gene Network database^39^, Phenotype and Genotype Integrator database, and GWAS Catalog^27^ ontology terms that are significantly enriched for (see Supplementary Data 3).

### GWAS literature, disease phenotypes, and eQTLs

We retrieved data from the previous GWAS of pulmonary function and pulmonary function-related phenotypes from GWAS Catalog^27^. This information was subset into two categories: “pulmonary reported”; for those studies that reported pulmonary function phenotype, and “pulmonary associated” for those that reported associations related to pulmonary function-related phenotypes (see Supplementary Data 4). We used the approach described above to identify variants previously reported to be associated with pulmonary function or pulmonary disease in GWA studies in the GWAS Catalog to identify novel variants associated with pulmonary function separately for Europeans and Africans. Briefly, for each variant we found associated with pulmonary function, we searched for variants in the GWAS Catalog that are in strong linkage disequilibrium (r^2^ > 0.4) with the variant. If any variant meets this criterion, we consider the associated variant in our study to have been previously reported elsewhere or otherwise novel. Furthermore, we obtained information on diseases associated with the genes in which the variants are located from the Pharos database^71^. Finally, information on SNPs that are expression quantitative trait loci in the lungs was obtained from the Genotype-Tissue Expression consortium database^72^.

### Statistics and reproducibility

We performed the statistical analyses in R programming language, MATLAB 2021a and Bash. We used the Welch test, Wilcoxon rank-sum test and the one-way analysis of variance to compare continuous measures among groups. All statistical tests were considered significant if the two-sided *p*-value was <0.05 for single comparisons. The multiple hypotheses tests were corrected by calculating a two-sided *q*-value (False Discovery Rate) for each group/comparison using the Benjamini and Hochberg procedure^73^.

### Reporting summary

Further information on research design is available in the Nature Portfolio Reporting Summary linked to this article.

## Supplementary information

Supplementary Information

Description of Additional Supplementary Files

Supplementary Data 1

Supplementary Data 2

Supplementary Data 3

Supplementary Data 4

Supplementary Data 5

Reporting Summary-New

## Data Availability

The datasets that support the results presented in this manuscript are available from: the UK Biobank; https://www.ukbiobank.ac.uk and https://pan-ukb-us-east-1.s3.amazonaws.com, dbSNP; https://www.ncbi.nlm.nih.gov/snp, the GWAS Catalog; https://www.ebi.ac.uk/gwas, and interactive Manhattan visualisations and regional plots of chromosomes found at https://public.tableau.com/app/profile/musalula.sinkala7788/viz/FEV1VariantsbyChromosome/ChromosomeFilter, https://public.tableau.com/app/profile/musalula.sinkala7788/viz/FVCVariantsbyChromosome/ChromosomeFilter, and https://public.tableau.com/app/profile/musalula.sinkala7788/viz/PEFVariantsbyChromosome/ChromosomeFilter. The source data underlying Figs. 1, 3, and 5 are presented in Supplementary Data 5. Furthermore, the GWA summary statistics derived by the Pan-UK Biobank project’s^64^ for the three pulmonary function parameters are available via the Amazon Web Services links: FCV: https://pan-ukb-us-east-1.s3.amazonaws.com/sumstats_flat_files/continuous-3062-both_sexes-irnt.tsv.bgz FEV1: https://pan-ukb-us-east-1.s3.amazonaws.com/sumstats_flat_files/continuous-3063-both_sexes-irnt.tsv.bgz PEF: https://pan-ukb-us-east-1.s3.amazonaws.com/sumstats_flat_files/continuous-3064-both_sexes-irnt.tsv.bgz.

## Code availability

Custom code written in MATLAB for processing and analysis of the data presented here is freely available at 10.5281/zenodo.7516618^74^.
