# Supplementary material for: A genome-wide association study identifies distinct variants associated with pulmonary function among European and African ancestries from the UK Biobank
Source: Commun Biol. 2023 Jan 14;6:49. doi: 10.1038/s42003-023-04443-8 (PMC9840173; doi:10.1038/s42003-023-04443-8)
Supplement: Supplementary file 1 — Supplementary Information [file 42003_2023_4443_MOESM1_ESM.pdf]

Supplementary Information

Supplementary Tables

Supplementary Table 1: Generalised linear regression model results

Statistics	Beta	Standard Error	t-statistics	p-value
FVC				
Intercept	3.99	0.09	44.2	$< 1 \times 10^{-300}$
Ethnicity (EUR)	0.81	0.09	8.9	5.15×10^{-19}
Age	-0.034	0.0017	-19.26	1×10^{-82}
Ethnicity (EUR): Age	-0.00087	0.00175	-0.497	0.619
FEV1				
Intercept	4.98	0.1226	40.65	$< 1 \times 10^{-300}$
Ethnicity (EUR)	0.858	0.1230	6.970	3.17×10^{-12}
Age	-0.0397	0.0023	-16.68	1.88×10^{-62}
Ethnicity (EUR): Age	0.002276	0.0023	1.156	0.2475
PEF				
Intercept	522.75	15.974	32.73	1.32×10^{-234}
Ethnicity (EUR)	94.32	16.039	5.881	4.08×10^{-9}
Age	-3.695	0.3102	-11.91	1.01×10^{-32}
Ethnicity (EUR): Age	-0.2962	0.3112	-1.952	0.341

Supplementary Figures

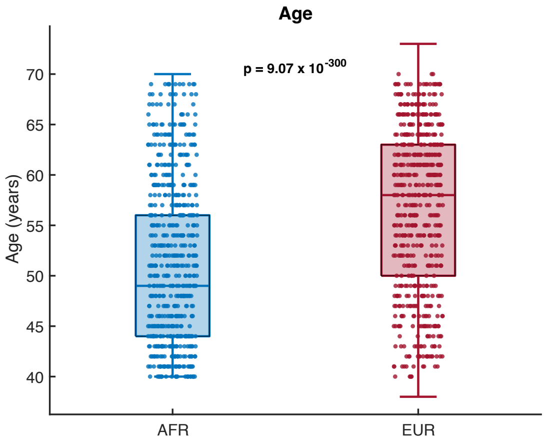

Supplementary Figure 1: *Comparison of the participant mean age at recruitment among Africans (n = 5,978) and Europeans (n = 383,471). The p-values shown for each comparison were calculated from Welch's t-test. On each box, the central mark indicates the median, and the left and right edges of the box indicate the 25th and 75th percentiles, respectively. The whiskers extend to the most extreme data points not considered outliers. To make the visualisation clearer, the filled circle mark showing the distribution only includes 1000 randomly sampled points from the total sample size of each group.*

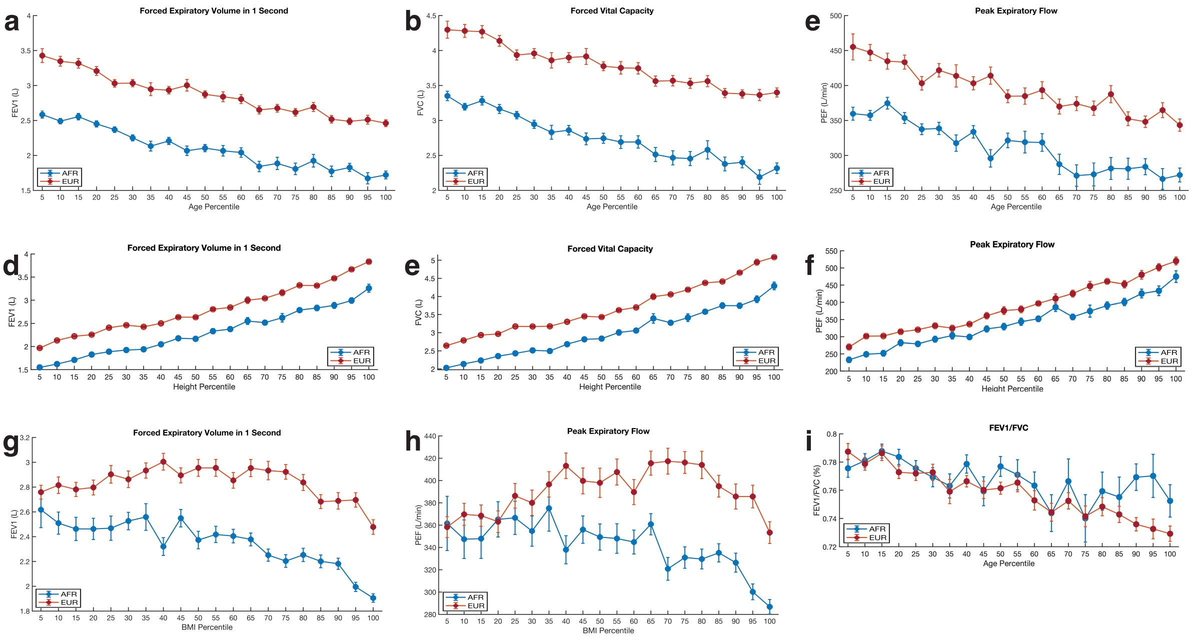

Supplementary Figure 2: *Relationship between Pulmonary function parameters to some anthropometric measurements. The error bars show the variation in* ***(a)*** *FEV1,* ***(b)*** *FVC and* ***(c)*** *PEF across age percentiles among Africans* (n = 5,978) *and Europeans* (n = 383,471). *Variations in* ***(e)*** *FEV1,* ***(f)*** *FVC and* ***(g)*** *PEF across height percentiles among Africans and Europeans.* ***(h)*** *FEV1 and* ***(i)*** *PEF across BMI percentiles and* ***(j)*** *FEV1/FVC across age percentiles. The middle point indicates the mean parameter value, and the error bars indicate the standard error of the mean at a particular height, age, or BMI percentile.*

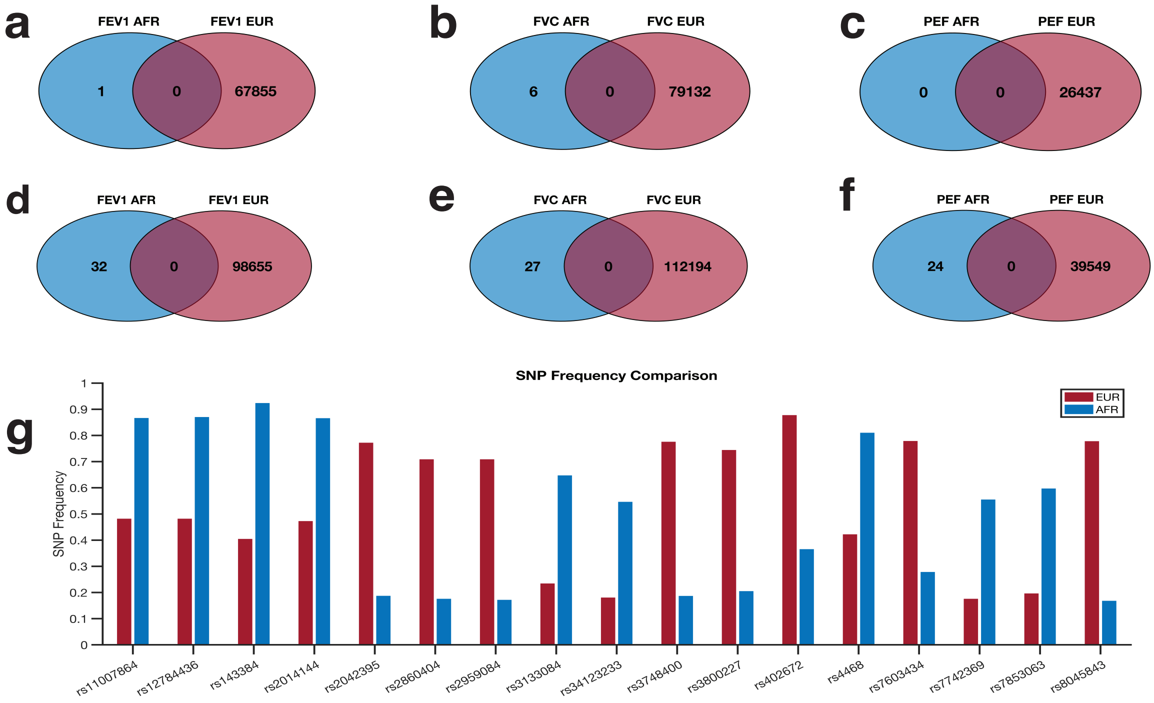

Supplementary Figure 3: *The Venn diagrams showing the distribution of genetic variants the significant (p-value of 5 x 10*$^{-8}$*, irrespective of the linkage disequilibrium and causal probability) variants associated with* ***(a)****, FEV1* ***(b)****, FVC, and* ***(e)*** *PEF in Africans and Europeans. The Venn diagrams show the distribution of the significant SNPs associated with* ***(d)****, FEV1* ***(e)****, FVC, and* ***(f)*** *PEF in Africans and Europeans based on the suggestive p-value of 1 x 10*$^{-6}$*.* ***(g)*** *Bar graph showing the SNPs associated with pulmonary function that exhibit the most significant difference in*

frequencies among Africans and Europeans. These are not necessarily the most likely predicted causal variants associated with pulmonary function among individuals and African and European Ancestry. Refer to Supplementary File 1 for details concerning individual SNPs and their frequencies among Africans and Europeans.

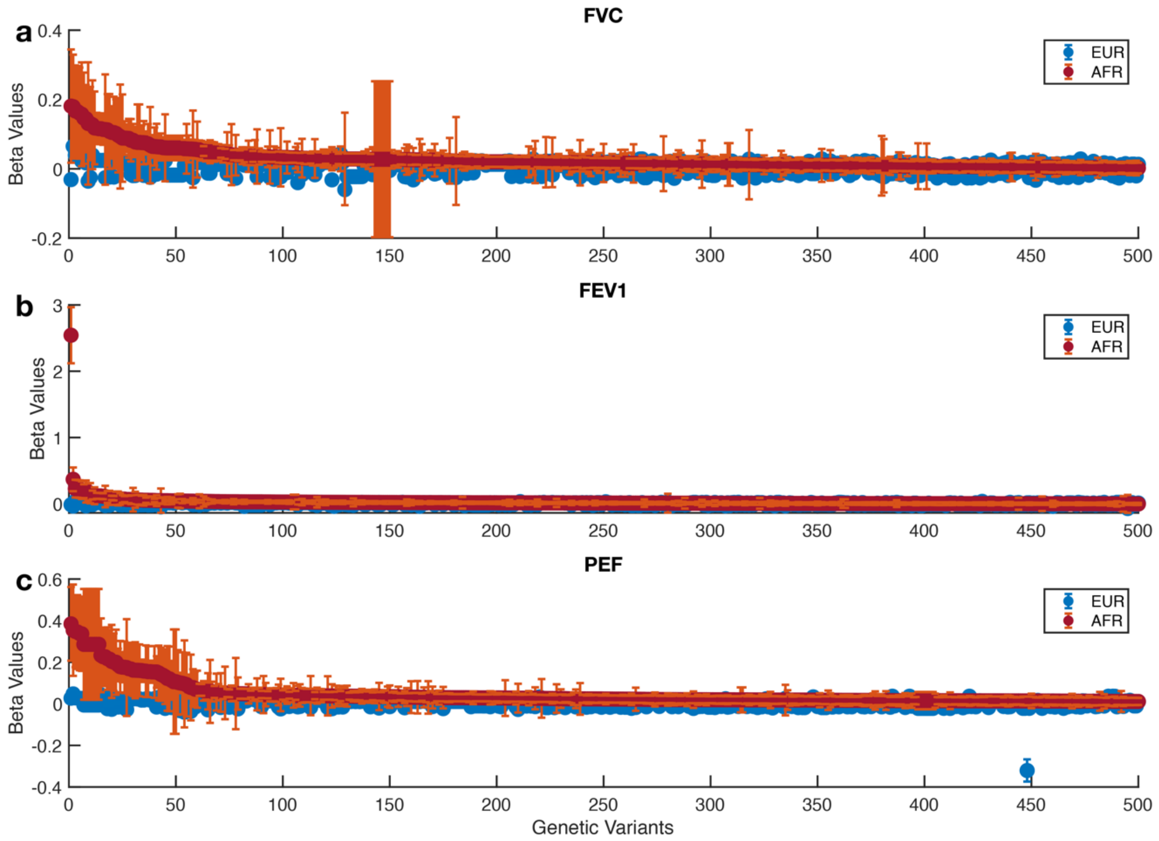

Supplementary Figure 4: *The top 500 most significant variants in African individuals associated with (a) FVC, (b) FEV1, and (c) PEF. The y-axis shows the beta estimates of the GWA calculated using the Generalised mixed model approach. The middle point of the 500 error bars shows the beta estimates, and the whiskers show the associated standard error of the beta estimates. Again, the makers are coloured based on ethnicity.*

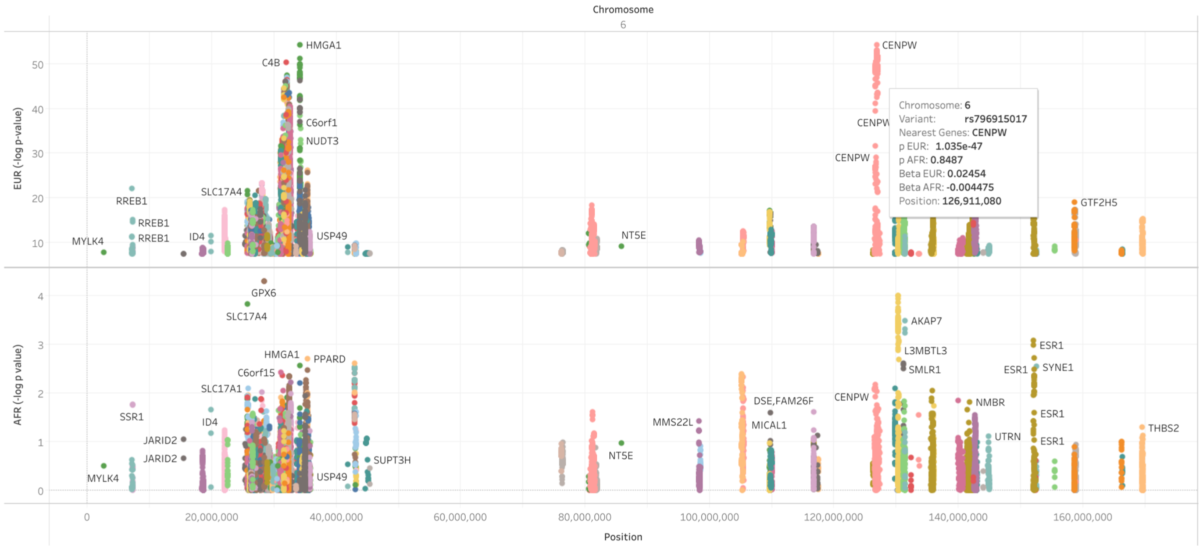

Supplementary Figure 5: *Screenshot of the interactive Manhattan visualisation for comparing variants on chromosome 6 associated with FVC in Europeans and Africans found [here](). The colour shows details of individual genes. Moving the cursor over a plotted variant displayed essential details about the variant, including the nearest gene (s), variant ID, GWA beta estimates in the Africans and Europeans, GWA p-value in the Africans and Europeans, and the position of the variant.*

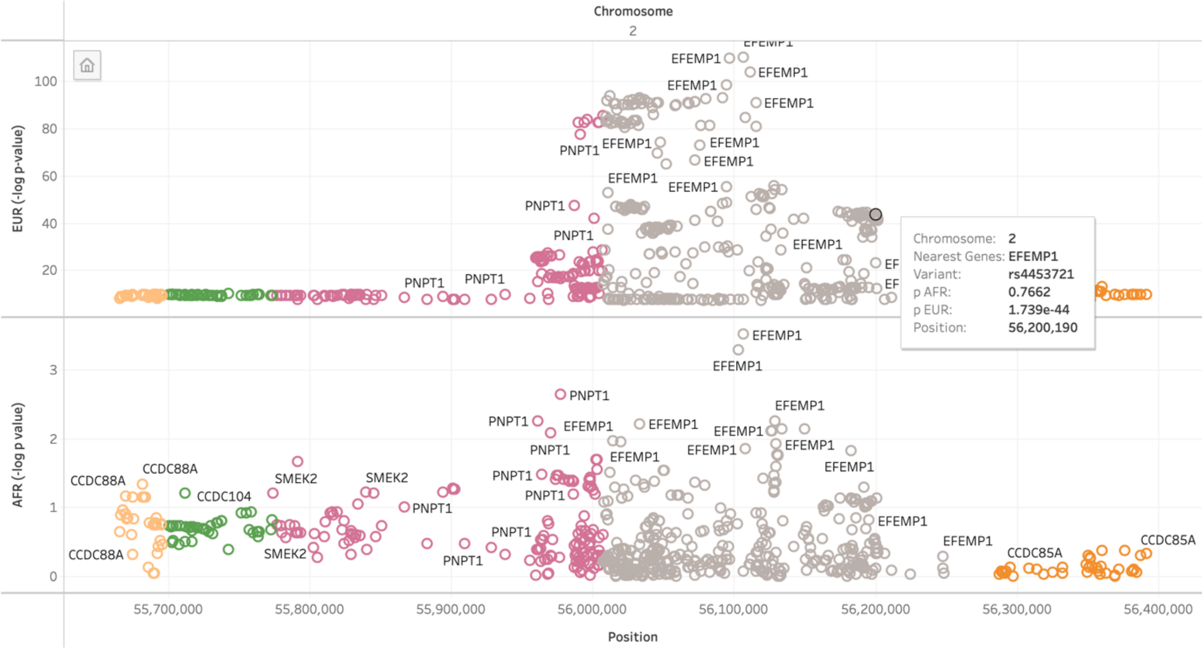

Supplementary Figure 6: *Screenshot of the interactive Manhattan visualisation for comparing variants on chromosome 2 associated with FVC in Europeans and Africans. The colour shows details of individual genes. The figure shows a view of chromosome 2 after zooming into the region between positions 55,700,000 and 56,400,000 to review variants associated with FCV near the genes PNPT1 and EFEMP1.*

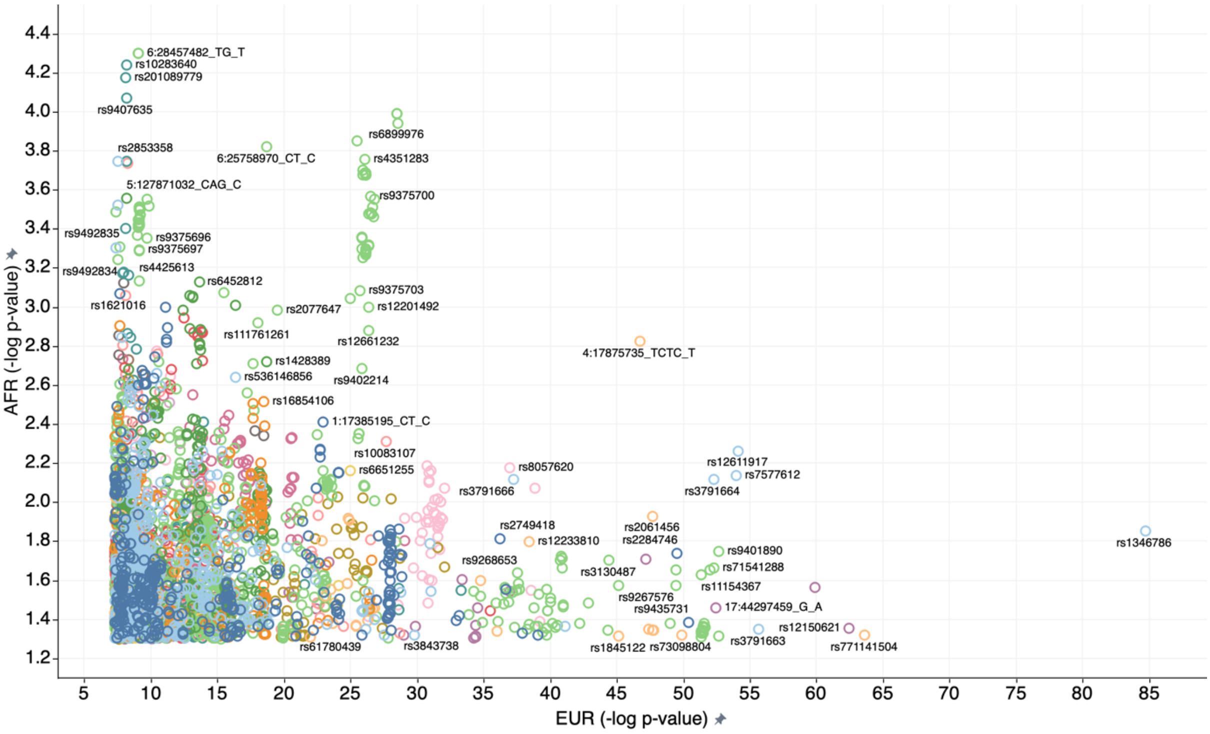

Supplementary Figure 7: *Variants significantly associated with FVC. The scatter plot shows the -log p-value in Europeans vs -log p-value in AFR individuals. The colour shows details about chromosomes. The marks are labelled by variant ID. The plotted data is filtered to keep only significant variants at a GWA threshold of 5 x10-8 in the EUR group and 0.05 in the AFR group.*

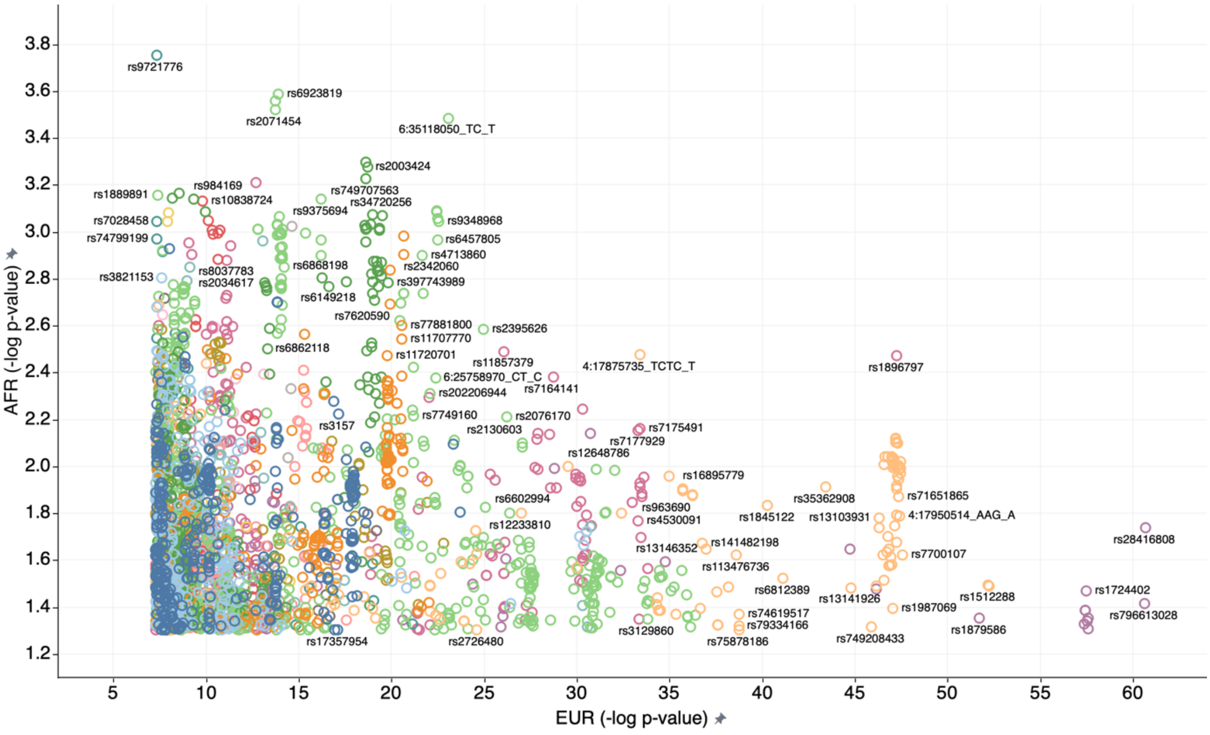

Supplementary Figure 8: *Variants significantly associated with FEV1. The scatter plot shows the -log p-value in Europeans vs the -log p-value in Africans. The colour shows details about chromosomes. The marks are labelled by variant ID. The plotted data is filtered to keep only significant variants at a GWA threshold of 5 x10-8 in the Europeans and 0.05 in the Africans.*

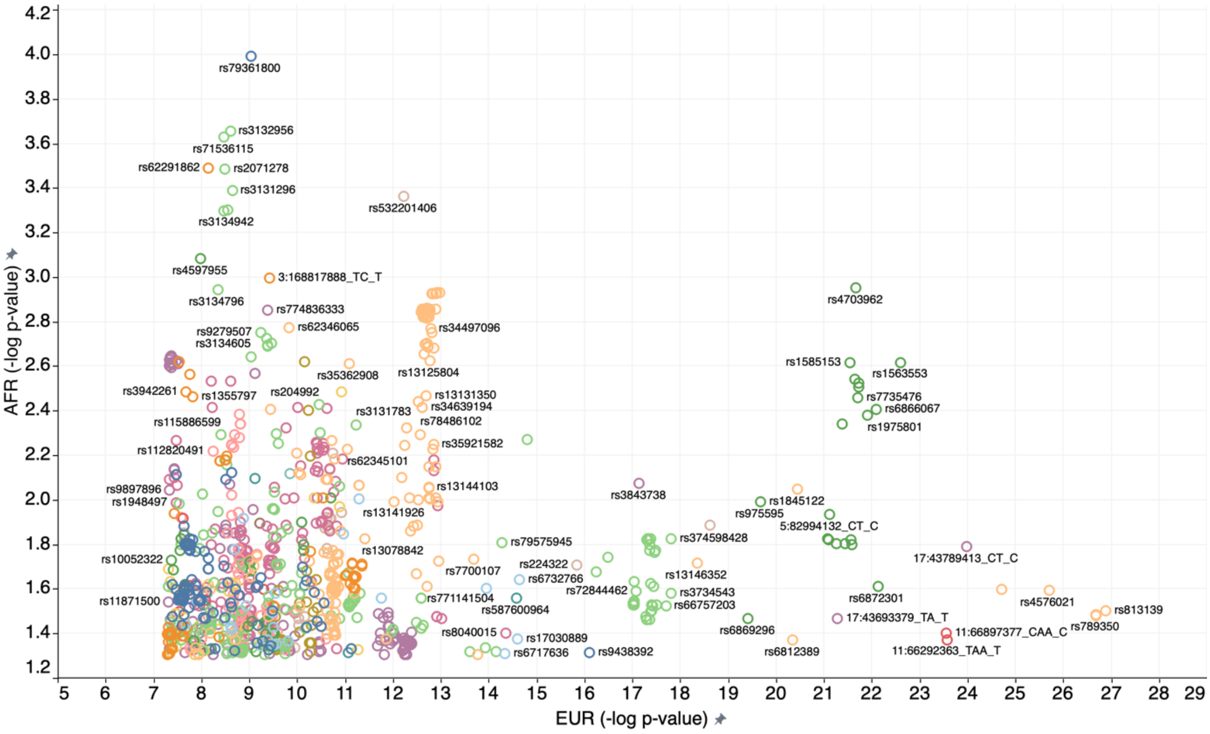

Supplementary Figure 9: *Variants significantly associated with PEF. The scatter plot shows the -log p-value in Europeans vs the -log p-value in Africans. The colour shows details about chromosomes. The marks are labelled by variant ID. The plotted data is filtered to keep only significant variants at a GWA threshold of 5 x10^{-8} in the Europeans and 0.05 in the Africans.*

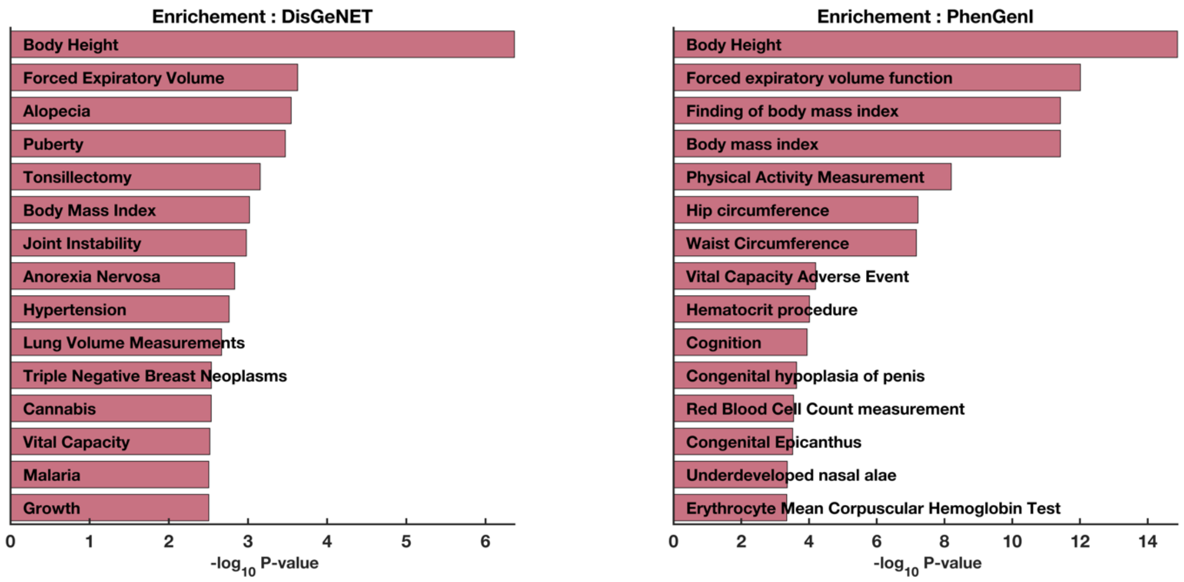

Supplementary Figure 10: *Enrichment analysis results using the genes in which the novel SNPs are located based on the Disease Gene Interaction Network, and Phenotype and Genotype Integrator databases.*

Supplementary Notes

Supplementary Note 1: Impact of 5-year difference on pulmonary function measures

We assessed the mean age of European (N = 383,471) and African (N = 5,978) participants in the UK Biobank datasets. We found that the mean age was significantly higher for the Europeans (mean = 56.8 years) compared to the Africans (mean = 51 years) (Welch test: $t = -41.3$, p = 9.07×10^{-300}; Supplementary Figure 1).

Next, we assessed whether the observed mean difference in the FVC, FEV1, and PEF among individuals of African and European ancestry is driven by the observed age difference among the two comparison groups. Here, we observed that for both the FVC and FEV1, the 90th percentile measures of Europeans were higher than the 10th age percentile of Africans (Supplementary Figures 2a and 2b). Furthermore, we observed that for PEF, the 50th percentile measure of Europeans was higher than the 10th age percentile of Africans (Supplementary Figure 2c).

Furthermore, we applied a Generalised Linear Model[1] with the assumption that measures are normally distributed to assess the interaction between age with ethnicity and the impact of both age and ethnicity on the pulmonary function measures. Our results reveal significant model for FVC ($F = 2.03 \times 10^4$, $p < 1 \times 10^{-300}$), FEV1 ($F = 1.28 \times 10^4$, $p < 1 \times 10^{-300}$), and PEF ($F = 5.86 \times 10^3$, $p < 1 \times 10^{-300}$), see Supplementary Table 1.

The generalised linear model results showed that the coefficient for the interaction between ethnicity (Europeans versus Africans) and age is not significant for FVC (p = 0.619), FEV1 (p = 0.247), and PEF (p = 0.341). Moreover, the large *p*-values (> 0.05) for all the interaction tests across pulmonary function measures indicate that the model specification might not differ statistically from a constant model. Therefore, we conclude that the observed higher FVC ($t = 8.9$, p = 5.15×10^{-19}), FEV1 ($t = 6.97$, p = 3.17×10^{-12}), and PEF($t = 5.88$, p = 4.08×10^{-9}), in Europeans, compared to Africans is not due to the age difference between the two groups, even though the

FVC (t = -19.26, p = 1.0 x 10^{-82}), FEV1 (t = -16.68, p = 1.88 x 10^{-62}), and PEF (t = -11.91, p = 1.01 x 10^{-32}), tend to reduce with age (Supplementary Table 1).

Supplementary Note 2: Comparison of variants associated with pulmonary function

Since the SNPs significantly associated with pulmonary function were unique for Europeans and Africans, we next set to compare the estimated beta value for all SNPs with a GWA significance of < 0.05. Here, we found that the most statistically significant SNPs in AFRs had relatively larger beta estimates in Africans than in Europeans for the FCV, FEV1, and PEF (Supplementary Figure 4). Overall, this finding showed that the SNPs significantly associated with pulmonary function in Africans demonstrate larger effect sizes than in Europeans.

We found that thousands of SNPs in Europeans were significantly associated with pulmonary function measures at the cut-off threshold of 0.05. Therefore, we found it challenging to visualise the top-ranking SNPs on statics scatter plots. Furthermore, we found it complicated to visualise and compare the significance of SNPs at various linkage disequilibrium blocks. Therefore, we used Tableau online software to create interactive Manhattan visualisations to allow an unbiased comparison of all variants associated with pulmonary function among Africans and Europeans. We only include SNPs with p-values below the 0.05 cut-off threshold for Europeans or Africans. These interactive Manhattan visualisations for SNPs associated with each pulmonary function measure can be found at the following links:

1. https://public.tableau.com/app/profile/musalula.sinkala7788/viz/FEV1VariantsbyChromosome/ChromosomeFilter
2. https://public.tableau.com/app/profile/musalula.sinkala7788/viz/FVCVariantsbyChromosome/ChromosomeFilter
3. https://public.tableau.com/app/profile/musalula.sinkala7788/viz/PEFVariantsbyChromosome/ChromosomeFilter

The interactive Manhattan plots allow for the following:

1. Comparative visualisation of all the significant variants associated with pulmonary function in Europeans or Africans across the 22 chromosomes.
2. Navigating a particular chromosomal location to compare African and European variants (Supplementary Figure 5).
3. Compare variants within or near specific genes by filtering the visualisation using a gene name.
4. Compare variants between Europeans and Africans at specific linkage disequilibrium blocks (Supplementary Figure 6).
5. Filtering variants in the visualisation using arbitrary p-value cut-off points to aid fine-grained comparisons.
6. Comparing the effect size of genetic variants associated with pulmonary function using the calculated beta estimates.

Supplementary Note 3: Replication of significant findings at a lowered significance level

Since we found that the variants associated ($p < 5 \times 10^{-8}$) with pulmonary function in Africans were not associated with pulmonary function in Europeans, we attempted to replicate the significant variants at a reduced p-value threshold of 0.05. We found that none of the six variants significantly associated with FVC in Africans had p-values less than 0.05 in Europeans. Furthermore, we found no other variants in Europeans that were in strong linkage disequilibrium with the significant variants in Africans. In addition, None of the significant variants in Africans exhibited a p-value less than 0.05 in Europeans (refer to the visualisation here). Overall, our results show that the variants we found associated with pulmonary function in Africans are likely only associated with this trait in Africans.

Furthermore, we aimed to replicate variants associated ($p < 5 \times 10^{-8}$) with pulmonary function in Europeans, in Africans at a p-value of less than 0.05. Interestingly, among the 79,132 variants associated with FVC in Europeans, we found 3,958 (5.0%) associated with FVC in Africans at a p-value cut-off of 0.05. Furthermore, among the 67,855 variants associated with FEV1 in Europeans, we found 3,360 (4.95%) associated with FEV1. Finally, 972 (3.7%) of the 26,437 variants associated with

PEF in Europeans were associated with pulmonary function in Africans at the p-value cut-off threshold of 0.05.

It should be noted that:

- The percentage of variants (~5%) we found associated with pulmonary function parameters at the 5% significance levels is expected by chance considering the number of the hypotheses tested here.
- Unlike in our analysis of replicating variants in Africans that are significant in Europeans, we have not considered these variants' linkage disequilibrium correlation coefficients.
- Here, our analysis includes all the significant variants (p-values $< 5 \times 10^{-8}$) in Europeans and not only the predicted causal variants within a particular linkage disequilibrium locus.

Therefore, we request the reader to examine these variants (or any of their interest) unbiasedly using the web resource we have provided, as highlighted previously.

We found several variants that could be associated with FVC in Europeans and Africans by comparing the calculated p-values of the two groups. These include, among others, two *GPX6* variants 6:28457482_TG_T (EUR = 8.12×10^{-10}, AFR = 5.02×10^{-5}), and rs80210770 (EUR = 8.12×10^{-10}, AFR = 5.02×10^{-5}). *SMARCA2* variants rs10283640 (EUR = 6.19×10^{-9}, AFR = 5.90×10^{-5}) and rs201089779 (EUR = 7.16×10^{-09}, AFR = 6.68×10^{-5}). *L3MBTL3* variants rs9388767 (EUR = 3.0×10^{-29}, AFR = 1.02×10^{-4}), refer to Supplementary Figure 7, the interactive visualisation here, and Supplementary Data 5.

Furthermore, we found several variants that could be associated with FEV1 in Africans based on the observed p-values. These include, among others, the *SMARCA2* variants rs7028458 (EUR = 4.47×10^{-08}, AFR = $1 \times 9.07 \times 10^{-4}$) and rs9721776 (EUR = 4.67×10^{-08} and AFR = 1.77×10^{-4}), *L3MBTL3* variants rs6923819 (EUR = 1.26×10^{-14}, AFR = 2.59×10^{-4}) and rs775280962 (EUR = 1.70×10^{-14}, AFR = 2.76×10^{-4}), and *CEP120* variant rs35348432 (EUR = 2.25×10^{-19}, AFR

= 5.05 x 10^{-4}). See Supplementary Figure 8, Supplementary Files 5 for the top-30 ranked variants and the interactive visualisation here.

We found 972 variants among 26,437 variants associated with PEF in Europeans ($p < 5 \times 10^{-8}$) that may also be associated with PEF in Africans ($p < 0.05$). These include, *FAM132A* variants rs79361800 (EUR = 9.20 x 10^{-10}, AFR = 1.02 x 10^{-5}) and rs71536115 (EUR = 3.34 x 10^{-09}, AFR = 2.36 x 10^{-4}), *LIPH* variant rs62291862 (EUR = 7.08 x 10^{-09}, AFR = 3.24 x 10^{-5}), RBM39 variant rs532201406 (EUR = 5.79 x 10^{-13}, AFR = 4.36 x 10^{-5}, a *HTR4* variant rs4597955 (EUR = 1.04 x 10^{-8}, AFR = 8.25 x 10^{-5}). Supplementary Figure 9, the interactive visualisation here, and Supplementary Data 5.

Supplementary References

1. Dobson, A.J. *An introduction to generalised linear models*, vii, 225 p. (Chapman & Hall/CRC, Boca Raton, 2002).
