# Supplementary material for: A genome-wide association study identifies distinct variants associated with pulmonary function among European and African ancestries from the UK Biobank
Source: Commun Biol. 2023 Jan 14;6:49. doi: 10.1038/s42003-023-04443-8 (PMC9840173; doi:10.1038/s42003-023-04443-8)
Supplement: Supplementary file 2 — Description of Additional Supplementary Files [file 42003_2023_4443_MOESM2_ESM.docx]

# Description of Supplementary Data

**Supplementary Data 1:** Supplementary data of genome-wide associations with pulmonary function in the manuscript. The spreadsheet contains the following results/datasets according to the sheet name. ***FVC AFR:*** SNPs that are significantly associated with FVC in Africans. ***FVC EUR*:** SNPs that are significantly associated with FVC in Europeans. ***FEV1 AFR:*** SNPs that are significantly associated with FEV1 in Africans. ***FEV1 EUR*:** SNPs that are significantly associated with FEV1 in Europeans. ***PEF AFR:*** SNPs that are significantly associated with PEF in Africans. ***PEF EUR*:** SNPs significantly associated with PEF in Europeans.

**Supplementary Data 2:** Frequency of genome-wide associated SNPs with pulmonary function among Africans and Europeans: The spreadsheet contains the following results/datasets according to the sheet name. ***SNP Freq – Annon;*** Frequency of SNPs in Africans and Europeans and the related p-value of the frequency difference calculated using the Fisher exact test. The sheet also contains information on which the phenotype(s) associated with the particular SNP. ***Location of SNPs;*** Gene within which the significant SNPs are located for Africans and Europeans.

**Supplementary Data 3:** GWAS enrichment analyses: The spreadsheet contains the following results/datasets according to the sheet name. ***GWAS Catalog-EUR;*** GWAS catalogue terms that we found significantly enriched in Europeans based on the genes in which the genome-wide significant SNPs associated with pulmonary function are located. ***GWAS Catalog-AFR;*** GWAS catalogue terms that we found significantly enriched in Africans based on the genes in which the genome-wide significant SNPs associated with pulmonary function are located.

**Supplementary Data 4:** Classification of causal variants associated with pulmonary function: The spreadsheet contains the following results/datasets according to the sheet name. ***PulmonaryReported;*** SNPs reported in GWAS catalogue to be associated with pulmonary function. ***PulmonaryAssociated;*** SNPs related to phenotypes correlated to pulmonary function (e.g., height, see Supplementary Figure 1). ***LungDiseaseAssociated;*** SNPs that fall within genes reported to be associated with pulmonary function and/or disease. ***eQTL;*** SNPs that are eQTLs in the lung as reported by the GTEx project. ***Novel;*** the novels SNPs, i.e., that do not meet any of the previously listed criteria. ***Lead SNPs;*** the top-lead SNPs that are found associated with pulmonary function in Europeans and Africans.

**Supplementary Data 5:** source data underlying the graphs and charts presented in the main figures in the manuscript. The spreadsheet contains the following datasets according to the sheet name. ***Fig 1 Source Data***: data to reproduce Figure 1 panels. ***Fig 3a Source Data***: data to reproduce Figure 3a. ***Fig 3b Source Data:*** data to reproduce Figure 3b. ***Fig 5 Source data:*** data reproduce Figure 5 panels.
